# Screening of *Saccharomyces cerevisiae* metabolite transporters by ^13^C isotope substrate labeling

**DOI:** 10.3389/fmicb.2023.1286597

**Published:** 2023-11-27

**Authors:** Lyubomir Dimitrov Stanchev, Iben Møller-Hansen, Pawel Lojko, Catarina Rocha, Irina Borodina

**Affiliations:** The Novo Nordisk Foundation Center for Biosustainability, Technical University of Denmark, Kongens Lyngby, Denmark

**Keywords:** transport proteins, *Saccharomyces cerevisiae*, metabolite transport, LC–MS/MS, amino acids, ^13^C isotopic labeling, screening assay

## Abstract

The transportome of *Saccharomyces cerevisiae* comprises approximately 340 membrane-bound proteins, of which very few are well-characterized. Elucidating transporter proteins’ function is essential not only for understanding central cellular processes in metabolite exchange with the external milieu but also for optimizing the production of value-added compounds in microbial cell factories. Here, we describe the application of ^13^C-labeled stable isotopes and detection by targeted LC–MS/MS as a screening tool for identifying *Saccharomyces cerevisiae* metabolite transporters. We compare the transport assay’s sensitivity, reproducibility, and accuracy in yeast transporter mutant cell lines and *Xenopus* oocytes. As proof of principle, we analyzed the transport profiles of five yeast amino acid transporters. We first cultured yeast transporter deletion or overexpression mutants on uniformly labeled ^13^C-glucose and then screened their ability to facilitate the uptake or export of an unlabeled pool of amino acids. Individual transporters were further studied by heterologous expression in *Xenopus* oocytes, followed by an uptake assay with ^13^C labeled yeast extract. Uptake assays in *Xenopus* oocytes showed higher reproducibility and accuracy. Although having lower accuracy, the results from *S. cerevisiae* indicated the system’s potential for initial high-throughput screening for native metabolite transporters. We partially confirmed previously reported substrates for all five amino acid transporters. In addition, we propose broader substrate specificity for two of the transporter proteins. The method presented here demonstrates the application of a comprehensive screening platform for the knowledge expansion of the transporter-substrate relationship for native metabolites in *S. cerevisiae.*

## Introduction

Transporting metabolites and molecules across biological membranes is a fundamental cellular process that ensures the proper functioning and growth of both prokaryotic and eukaryotic organisms. Small non-ionized molecules such as gasses passively diffuse biological membranes (Yang and Hinner, 2015). Most metabolites and more complex molecules are transported via specialized membrane-bound proteins, namely membrane transporters (Saier, 2000; Kell and Oliver, 2014). Despite their crucial importance for the functioning of the cells, membrane transporters remain one of the most under-characterized protein groups (César-Razquin et al., 2015). According to TransportDB and Transporter Classification Database (Elbourne et al., 2017; Saier et al., 2021), the genome of the model organism *Saccharomyces cerevisiae* encodes 341 membrane transporters, with only a few of them being experimentally deorphanized.

Apart from their biological significance, the interest in metabolite transporters has been increasing in industrial biotechnology (Sauer, 2022). Transporters implicated in strain design strategies can allow the uptake of alternative substrates, prevent leakage of pathway intermediates, and modulate the export of the desired product (Borodina, 2019; van der Hoek and Borodina, 2020). A prominent example is the engineering of *S. cerevisiae* for the production of ethanol, which was greatly improved by coupling the Gal2 xylose importer to a xylose isomerase from *Clostridium phytofermentans* (Thomik et al., 2017). Another notable example is the engineering and overexpression of the Hxt transporters that led to improved co-consumption of sugars for isobutanol production in yeast (Promdonkoy et al., 2019). Moreover, transporter overexpression enhanced the export and, thereby, the production of lactic acid as demonstrated by several studies (Casal et al., 1999; Soares-Silva et al., 2003; Branduardi et al., 2006; Pacheco et al., 2012; Mans et al., 2017). However, using transporter engineering as a tool for optimizing cell factories remains a limited tool, mainly due to the extensive knowledge gaps about transporters’ function and their respective substrates (van der Hoek and Borodina, 2020; Belew et al., 2022). At present, *in vivo* assays employed for identifying and characterizing membrane transporters predominantly depend on phenotypes with readily interpretable outcomes or involve incubation with labeled substrates such as radiolabeled compounds (e.g., ^14^C or ^3^H). Yet, using radiolabeled substrates increases assay costs and requires specialized facilities. Another disadvantage of the radiolabeled substrates is the detection method by scintillation counting, which is mainly limited to ^3^H, ^14^C, or ^32^P commercially available radiolabeled substrates.

Current methodologies do not meet the requirements of industrial biotechnology for swift and accurate identification of hundreds of transporters or are limited to only the toxic plethora of compounds. Several metabolomics approaches based on mass-spectrometry have been successfully adapted for transport assay to overcome this bottleneck. A recent study in mammalian cells from Wright Muelas et al. (2020) describes a novel high-throughput strategy based on untargeted LC–MS/MS for identifying differential uptake patterns between mammalian cell lines.

Available high-throughput methods for transporter screening in *S. cerevisiae* have limited application to compounds leading to phenotypic change. Based on libraries of strains with deletion or overexpression of transporters, these assays depend on the mutant’s tolerance toward a panel of screened compounds. For instance, Lanthaler et al. (2011) screened a genome-wide transporter deletion library in *S. cerevisiae* and identified the carriers for 18 toxic compounds. In more recent work, Almeida et al. (2021) generated a double deletion transporter library of 122 plasma membrane transporters. They successfully applied it to discover possible export and import routes for 44 drugs.

Several expression platforms for studying transporters have been established throughout the years. Notably, among the *in vivo* transporter expression systems, *Xenopus laevis* oocytes excel in several features. *Xenopus* oocytes system has negligible background transport activity, allows both import and export transporter assays, possesses the capability of transporter protein synthesis, and has a low codon bias. Moreover, robotic nano-injections allow the high-throughput screening of transporters.

We aimed to develop a metabolomics-based, high-throughput assay for identifying *S. cerevisiae* metabolite transporters and their corresponding substrates. We combine ^13^C metabolite labeling and targeted LC–MS/MS analysis to investigate yeast transporters *in vivo* for increased sensitivity. The amino acid transporters in *S. cerevisiae* are a well-characterized group of metabolite transporters with established knowledge regarding substrate specificity, membrane localization, and regulatory mechanisms (Bianchi et al., 2019). Our preference for amino acid transporters over other metabolite transporters, such as sugar transporters, was also guided by the objective of decoupling transport events from growth dependencies. Additionally, our selected LC–MS/MS method excels in detecting 15 amino acids but is less proficient in identifying sugars and organic acids. Considering our method’s primary goal of simultaneously screening multiple metabolites, this choice aligned with our research objectives. Consequently, to establish the validity of our method, we focused on five known *S. cerevisiae* amino acid carriers with well-established substrate preferences. We examined their transport profiles using two *in vivo* platforms. Initially, we tested our approach on yeast mutants with single amino acid transporter deletions or overexpressions. These mutants were cultured in a medium supplemented with ^13^C-glucose, allowing for metabolite labeling. Subsequently, the ^13^C-labeled yeast cells were subjected to a transport assay using an unlabeled pool of amino acids. To complement our findings in *S. cerevisiae*, we heterologously expressed the five amino acid carriers in *Xenopus* oocytes and performed an uptake assay using ^13^C-labeled yeast metabolite extract. The *S. cerevisiae* transport assay results revealed significant alterations in intra- and extracellular amino acid levels upon deletion or overexpression of the respective amino acid carriers. Encouragingly, the transport assay conducted in *Xenopus* oocytes produced partially similar uptake profiles. Collectively, the outcomes from both the *S. cerevisiae* and *Xenopus* oocytes transport assays provide compelling evidence supporting the suitability of our metabolomics method for identifying metabolite transporters in *S. cerevisiae*.

## Materials and methods

### Materials

Cloning materials were obtained from New England Biolabs (Ipswich, MA, USA) or Thermo Scientific (Waltham, MA, USA). Eurofins Genomics (Ebersberg, Germany) provided gene sequencing service, and oligonucleotides were purchased from Integrated DNA Technologies (Leuven, Belgium). Uniformly labeled U-^13^C- D-glucose (99% purity, catalog # CLM-1396-5) and U-^13^C (98%) yeast metabolite extract from *Pichia pastoris* were purchased from Euriso-Top (Saarbrücken, Germany). Unless stated otherwise, all other materials were purchased from Sigma Aldrich (München, Germany).

### Strains, media, and growth conditions

Yeast knockout mutant strains with BY4741 (MATa; *his3Δ1; leu2Δ0; met15Δ0; ura3Δ0*) background were purchased from Open Biosystems (catalog # YSC1053). Overexpression strains were generated in the same parent strain. All knockout and overexpression strains used in this study are listed in Supplementary Table S1. For transport assays, yeast pre-cultures were inoculated in 2 mL YPD medium (20 g/L glucose, 20 g/L bacto-peptone, 10 g/L yeast extract) in 24 deep-well plates and grown for 24 h at 30°C and 250 rpm. Optical density at 600 nm (OD_600_) was then measured and adjusted to 0.02 in 2 mL synthetic dextrose (SD) medium (20 g/L ^13^C-glucose, 7 g/L yeast nitrogen base, histidine 2 g/L, leucine 3 g/L, methionine 2 g/L, uracil 2 g/L) and cultured for 21 h or until mid-logarithmic phase (OD_600_ = 0.6 ~ 0.8). For growth profile measurements, cells from a yeast pre-culture were inoculated at OD_600_ = 0.01 in 96 deep-well plates (Enzyscreen B.V., CR1424) containing 300 μL SD medium with natural glucose. The cultures were grown for 72 h at 30°C with 300 rpm agitation, and measurements were acquired every 20 min.

### Cloning and plasmid construction

*Escherichia coli* DH5α strain was used for plasmid amplification, and successful transformants were selected on LB plates with 100 mg/mL ampicillin. Cloning was performed according to the EasyClone MarkerFree method (Jessop-Fabre et al., 2016). For the generation of transporter overexpression strains, the genes of interest were amplified from genomic DNA extracted from the wild-type BY4741 strain and cloned under the control of a constitutive TEF promoter. All genes were integrated into site XII-2 of the chromosome. Transformation of yeast was performed according to the lithium acetate method (Gietz and Woods, 2002), and clones carrying integrated genes were selected on a solid YPD medium supplemented with 200 mg/L G418. Successful integration was confirmed by colony PCR. All oligonucleotides and plasmids are listed in Supplementary Tables S2, S3, respectively.

Cloning of transporter genes for cRNA expression in *Xenopus laevis* oocytes (Ecocyte Bioscience, Dortmund, Germany) was completed as previously described (Wang et al., 2021). Briefly, amino acid transporter genes were first cloned into pCfB5245 plasmid encoding for T7p-β-globin 5-UTR and β-globin 3-UTR (Darbani et al., 2016). The resulting plasmids were then used as a PCR template to amplify the cassette fragment for *in vitro* RNA synthesis. Capped RNAs for transporter expression in oocyte cells were then synthesized with a T7 mMESSAGE mMACHINE™ kit (Ambion). The quality of the cRNAs (> 500 ng/μL) was analyzed by Agilent 2100 Bioanalyzer (Agilent Technologies) before proceeding with oocyte injection.

### Plate-based transport assay

After reaching an OD_600_ of 0.6 ~ 0.8 in SD medium, cell cultures were harvested and washed two times with 2 mL phosphate-buffered saline (PBS; 130 mM NaCl, 2.6 mM KCl, 7 mM Na_2_HPO_4_, 1.2 mM KH_2_PO_4_, pH 7.4) at 1000 *g* for 5 min at 23°C. The cell pellets were then resuspended in 400 μL of amino acid (AA) mix (adenine 13.3 μM, p-aminobenzoic acid 58 μM, leucine 290 μM, alanine 85.4 μM, arginine 43.6 μM, asparagine 57.5 μM, aspartic acid 57.1 μM, cysteine 62.7 μM, glutamic acid 51.7 μM, glutamine 52.0 μM, glycine 100 μM, histidine 49 μM, myo-inositol 42.2 μM, isoleucine 57.9 μM, lysine 52 μM, methionine 50.9 μM, phenylalanine 46 μM, proline 66 μM, serine 72.3 μM, threonine 63.8 μM, tryptophan 37.2 μM, tyrosine 42 μM, uracil 67.8 μM, valine 64.9 μM) and incubated for 40 min at 30°C and 300 rpm agitation.

### Metabolite extraction

Cells incubated with the AA mix were transferred to a 96-well protein precipitation filter plate (Waters Corp, MA, USA), and extracellular metabolites were filtrated via positive pressure at 35 psi to a 2 mL 96 deep-well plate. The cell pellets were then washed twice with 300 μL PBS by applying positive pressure at 35 psi. To extract the intracellular metabolites, 200 μL boiling ethanol 75% (v/v) was added to the cell pellets. The filter plate was then vortexed for 1 min at 450 rpm and immediately incubated at 65°C for 15 min. Extracted intracellular metabolites were then filtered by applying positive pressure at 35 psi and collected in a 2 mL 96 deep-well plate. Residual metabolites were collected by applying three times 150 μL of boiling 75% (v/v) ethanol, positive pressure at 35 psi, and pulling together all elutions. Intracellular metabolic extracts were then dried in a vacuum Eppendorf Concentrator plus system equipped with a plate rotor for 6 h at 30°C. Dried intracellular extracts were stored at - 80°C and resuspended in 200 μL LC–MS/MS grade water before being subjected to analysis.

### Transport assay in *Xenopus* oocyte cells

Oocytes were injected with 50 nL of cRNA (400 ng/μL transporter cRNA and 100 ng/μL GFP cRNA) using an automated RoboInject (Multichannel System, Germany) and incubated for 3 days at 18°C. After 3 days of incubation, GFP-positive cells were selected and subsequently used for the uptake assay. A minimum of four oocyte cells per biological replicate were pre-incubated in a Kulori buffer pH 7.4 and then transferred to ^13^C-labeled *P. pastoris* yeast extract pH 7.4 for 1 h at room temperature. Cells were subsequently transferred to 4°C Kulori buffer pH 7.4 to stop the assay and washed three times in fresh Kulori buffer pH 7.4 before being disrupted with 100 μL ice-cold 50% (v/v) methanol. Cell lysates were incubated for 2 h at −20°C and then spun down to remove cell debris. Finally, 70 μL from the supernatant was mixed with 50 μL water before subjecting it to LC–MS/MS analysis.

### Liquid chromatography-mass spectrometry

Samples were reconstituted in water (HPLC grade) and analyzed on a UHPLC-ESI-QTrap-MS (UHPLC LC20/30 series, Shimadzu and QTrap, Sciex) using an XSelect HSS T3 XP column (2.1 × 150 mm, particle size 2.5 μm, Waters) as previously described (McCloskey et al., 2015). Data was acquired using the scheduled MRM algorithm in Analyst Software (Sciex). Post-run data analysis was performed in SciexOS Software (Sciex). Compounds were quantified using ^13^C metabolically labeled internal standards from *E. coli*. Linear regressions for quantification were based on peak height ratios. The lower limit of quantification (LLOQ) was defined at a peak height greater than 1e3 ion counts and a signal-to-noise ratio greater than 15 (McCloskey et al., 2015).

Binary solvent gradient (A: 10 mM Tributylamine, 10 mM Acetic Acid, 5% (v/v) MeOH, 2% (v/v) Isopropanol in water; and B: Isopropanol) was applied: 0 to 5 min, isocratic 100% A; 5 to 9 min, linear ramp to 98% A, 2% B; 9 to 9.5 min, linear ramp to 94% A, 6% B; 9.5 to 11.5 min, isocratic 94% A, 6% B; 11.5 to 12 min, linear ramp to 89% A, 11% B; 12 to 13.5 min, isocratic 89% A, 11% B; 13.5 to 15.5 min, linear ramp to 72% A, 28% B; 15.5 to 16.5 min, linear ramp to 47% A, 53% B; 16.5 to 22.5 min, isocratic 47% A, 53% B; 22.5 to 23 min, linear ramp to 100% A; 23 to 27 min, isocratic 100% A. The flow rate was 0.4 mL min − 1 from 0 to 15.5 min, 0.15 mL min − 1 from 15.5 to 23 min, and 0.4 mL min − 1 from 23 to 27 min. The injection volume was 10 μL. MS data was acquired from 0.4 to 23 min (McCloskey et al., 2015).

### Data analysis

**Figure 1:**
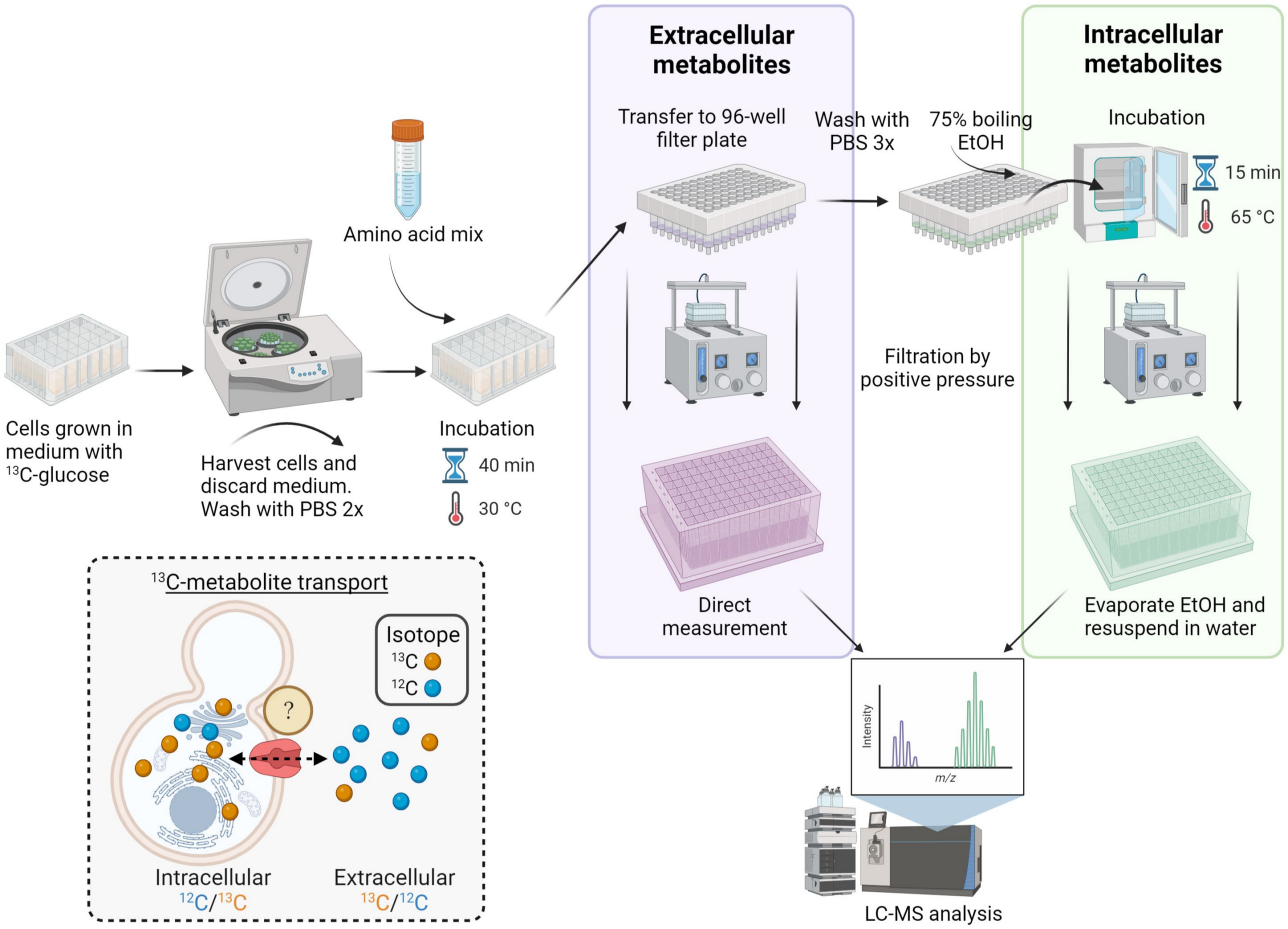
Workflow for high-throughput screening of *S. cerevisiae* membrane transporters. Yeast transporter overexpression or deletion mutant strains were grown in 24 deep-well plates in a medium containing U-^13^C-glucose and then harvested by centrifugation. Following two washing steps with PBS, the cells were incubated with an amino acid mix for 40 min at 30°C. The cell suspension was then transferred to a 96-well filter plate, and the extracellular metabolites were collected via positive pressure. The remaining cell pellets were washed two times with PBS and lysed by adding 75% boiling ethanol and incubated for 15 min at 65°C. After cell disruption, the intracellular metabolites were collected via a second round of filtration by positive pressure. Before LC–MS/MS analysis, ethanol was evaporated, and intracellular metabolites were resuspended in water. Inset depicts the *in vivo* transport assay of yeast cultured with U-^13^C-glucose (synthesizing ^13^C-amino acids; yellow spheres) and incubated with unlabeled (^12^C) amino acid mix (blue spheres). Transport of metabolites in the intracellular extract is calculated from the ratio of ^12^C/^13^C isotopes, whereas in the extracellular extracts, the ratio is ^13^C/^12^C.

For intracellular metabolite analysis, the spectral peak areas identified for the respective amino acids were normalized to the cell densities (OD_600_) of the background strain BY4741. The normalized peak area values were further used for isotopic ratio calculations. The targeted LC–MS/MS method allowed the detection of 102 metabolites, of which 15 were amino acids. The peak areas corresponding to the uniform ^12^C compound and its ^13^C analog were acquired for each amino acid. The transport of amino acids in the intracellular extract was determined by the ^12^C/^13^C ratio, whereas the ^13^C/^12^C ratio characterized transport in the extracellular extracts. Intracellular amino acid concentrations were calculated based on total amino acid concentrations from the intracellular extract and provided that the cell density of baker’s yeast is ~1.103 g/mL (Bryan et al., 2010). Analysis of growth profiles was performed with Python version 3.10.7. Data visualization was completed with OriginPro software version 9.8.0.200. Figures 1 and 5A were created with BioRender.com.

**Figure 2:**
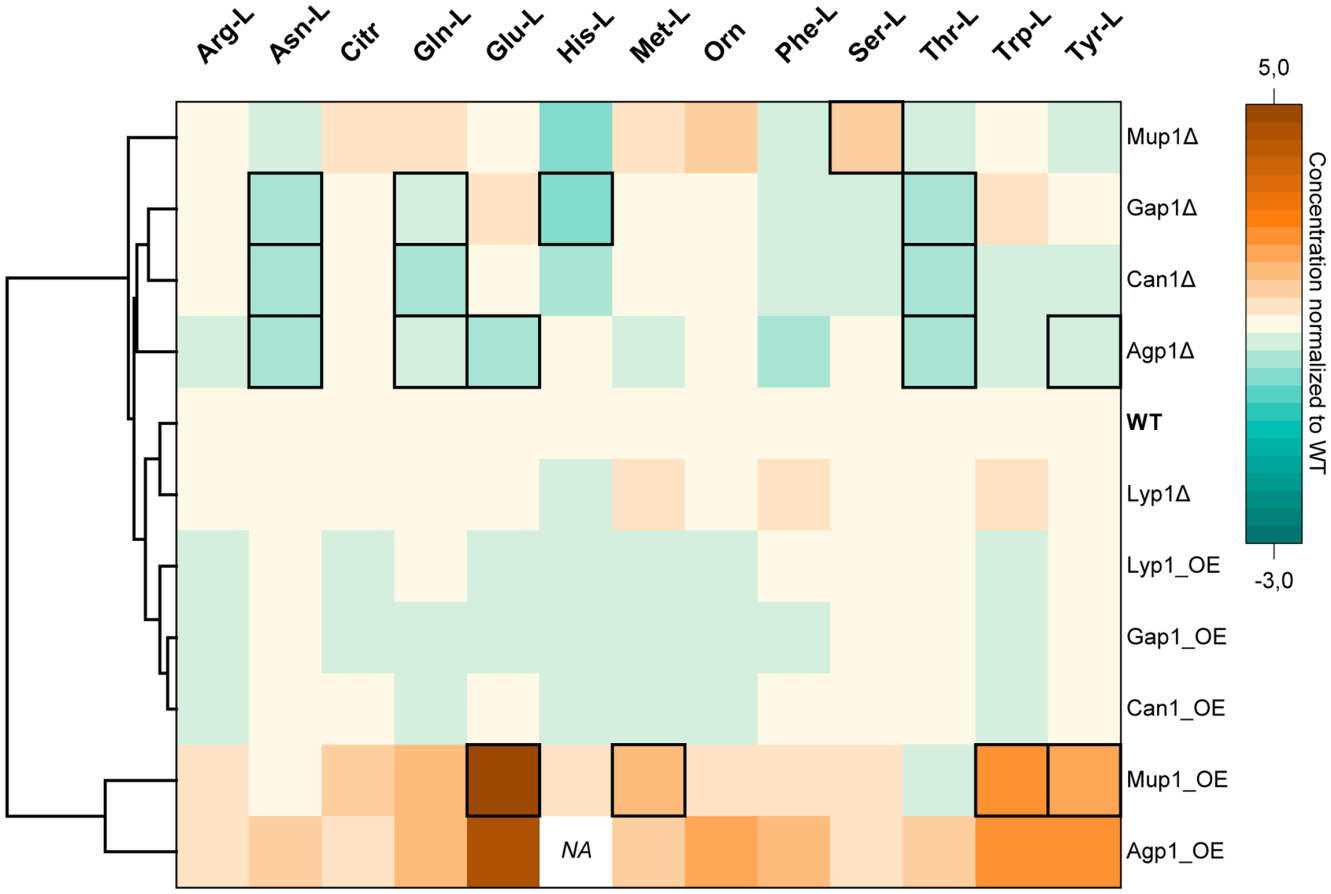
Hierarchical Clustering Heatmap of intracellular amino acid concentrations. The color code indicates the intracellular amino acid concentrations of transporter deletion (Δ) or overexpression (OE) mutants normalized to the values obtained for the wild-type (WT) yeast strain. The hierarchical clustering is based on the Euclidean distance of the average of all amino acids for each yeast cell line. Columns and rows correspond to amino acids and yeast transporter mutants, respectively. Data is the mean average of three biological replicates. Black frames denote statistically significant changes in concentrations (value of *p* < 0.05). Data point for His-L intracellular concentration in the Agp1 overexpression mutant was not detected (NA). (Absolute concentrations are available in Supplementary Table S4).

**Figure 3:**
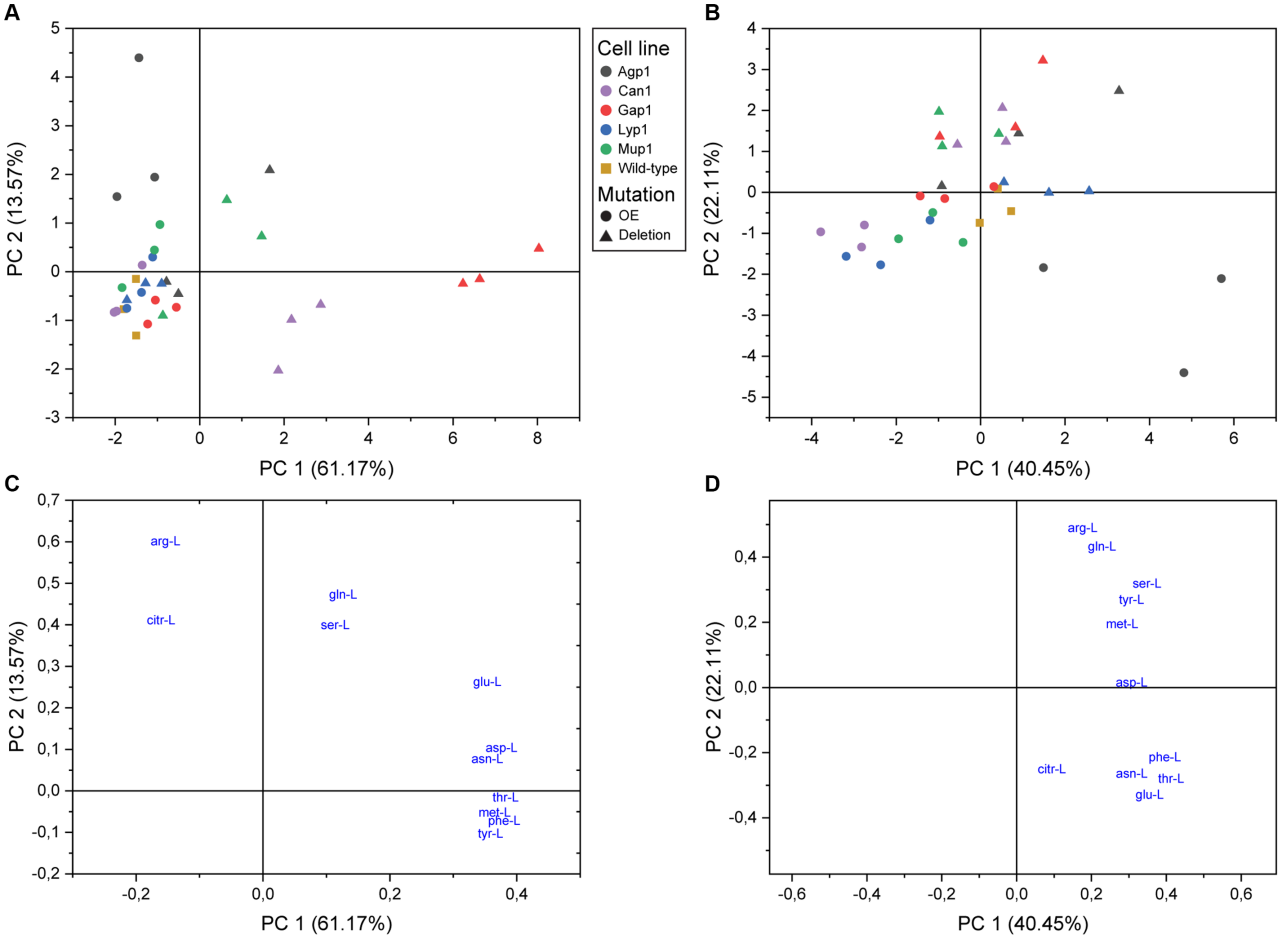
Principal component analysis of amino acid transport in *S. cerevisiae*. Score plots of intracellular **(A)** and extracellular **(B)** metabolite analysis. Yeast single transporter deletion mutants (Deletion; filled triangles), transporter overexpression strains (OE; filled circles), or wild-type yeast strain BY4741 (filled squares) were cultured in the presence of ^13^C-glucose until almost complete isotopic labeling. Subsequently, the transporter mutants were incubated with an unlabeled mix of amino acids and analyzed by LC–MS/MS. **(C,D)** Represent the loading plots with the 11 detected amino acids as variables for the intracellular and extracellular extracts, respectively.

**Figure 4:**
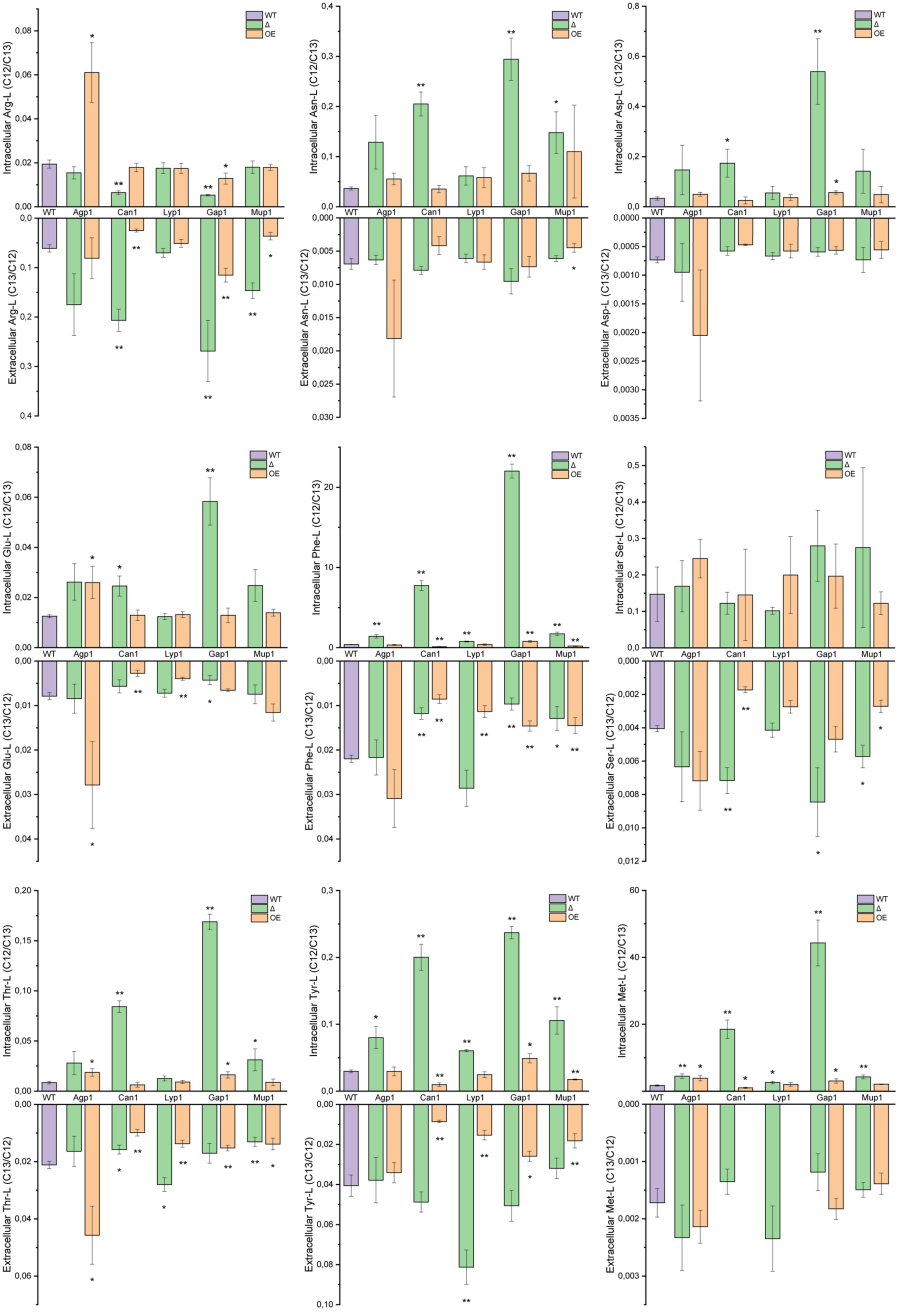
Transport of individual amino acids. Intra- and extracellular LC–MS/MS analysis of extracted amino acids. Yeast mutant strains with deleted or overexpressing Agp1, Can1, Gap1, Lyp1, or Mup1 amino acid transporters were grown in a medium containing ^13^C-glucose and subsequently incubated with unlabeled amino acid mix. The graphs represent the ratios of the peak areas for ^12^C and ^13^C single amino acids (^12^C/^13^C ratio for intracellular and ^13^C/^12^C for extracellular transport). Results are the average of three biological replicates with SD±. Extracellular Met-L for the Can1 and Lyp1 overexpression strains was not detected. Student *t*-test indicates a statistically significant difference compared to the wild-type strain. (* value of *p* < 0.05, ** value of *p* < 0.01). OE, overexpression strain; Δ, deletion mutant; WT, wild-type strain.

**Figure 5:**
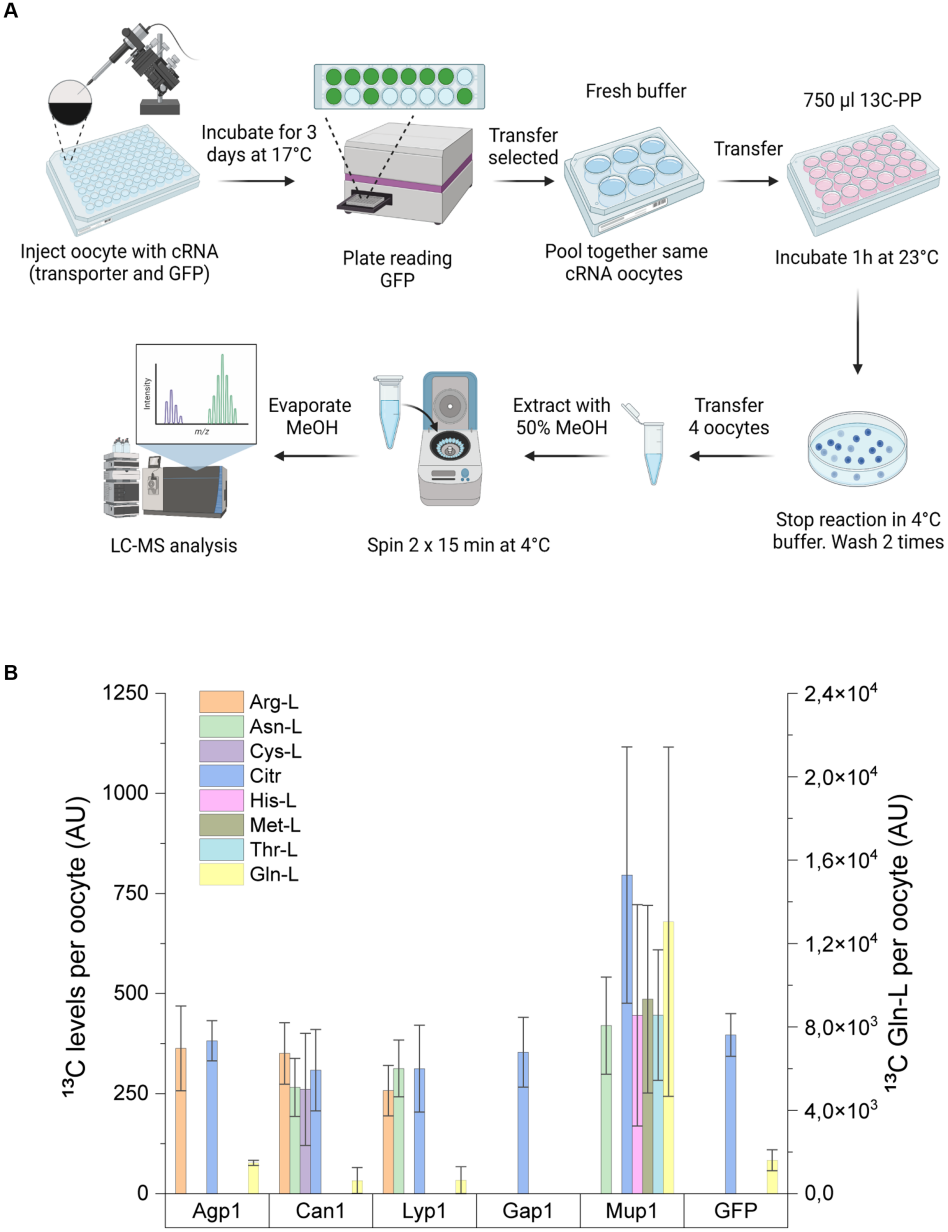
Amino acid uptake assay in *Xenopus* oocytes. **(A)** Workflow for uptake assay in *Xenopus* oocytes. *Xenopus* oocytes injected with cRNA encoding for GFP alone or in combination with Agp1, Can1, Lyp1, Gap1, or Mup1 were incubated for 3 days to allow the expression of transporters. GFP-positive oocytes injected with the same transporter protein were then pooled together and incubated with a uniformly labeled ^13^C yeast extract. The uptake assay was stopped after 1 h with a 4°C buffer, and oocytes were subsequently washed before extraction of the endo metabolites. The metabolite extracts were then subjected to LC-MS/MS analysis. **(B)** Analysis of ^13^C intracellular amino acid levels from *Xenopus* oocytes expressing single *S. cerevisiae* transporters or GFP. Right y-axis indicates the intracellular levels of ^13^C Gln-L. Results are the average of minimum of three biological replicates (each replicate consists of 3–4 oocytes) with SD±.

## Results

### Metabolomics strategy for the identification of metabolite transporters

Distinguishing between endogenous and exogenous metabolites is one of the main challenges in establishing an assay for transporter identification. Studies on intracellular metabolic fluxes have successfully taken advantage of stable isotope tracing for mapping biosynthetic pathways and distinguishing endogenous from exogenous metabolites (Zamboni et al., 2009). Utilizing a similar strategy, we decided to culture the yeast transporter mutants with uniformly labeled U-^13^C-glucose until reaching almost complete isotopic enrichment of endogenous metabolites after 21 h of cultivation (Wu et al., 2005; Figure 1).

After cultivation, the ^13^C labeled yeast cells were subjected to transport assay with a mixture of 20 proteinogenic unlabeled amino acids prior to targeted LC–MS/MS analysis (McCloskey et al., 2015). Analysis of both the intra- and extracellular metabolite extracts allowed us to monitor the alterations in the isotopologues ratio that served as an indicator of both transport occurrences and the operational direction of the transporter. Five *S. cerevisiae* amino acid transporters were selected to assess our method’s accuracy: Agp1, Can1, Gap1, Lyp1, and Mup1. All five transporters belong to the APC (amino acid-polyamine-organocation) family of transporters, according to the Transporter Classification Database TCDB 2.A.3 (Saier et al., 2021). Our criteria for transporter selection were based on the proteins’ localization at the plasma membrane and their known substrate ranges fitting to the metabolic extract. Agp1 and Gap1 represent amino acid transporters with a broad substrate specificity (more than seven amino acids), while Can1, Lyp1, and Mup1 have a relatively narrow range (less than four) of transported amino acids (Table 1).

**Table 1:** Amino acid transporters analyzed in this study.

**Name**	**Gene**	**Reported substrate**	**Reference**
Agp1	YCL025C	His, Asp., Glu, Ser, Thr, Asn (0.29 mM), Gln (0.79 mM), Cys, Gly, Pro, Ala, Val, Ile (0.6 mM), Leu (0.16 mM), Met, Phe (0.6 mM), Tyr, Trp	Schreve et al. (1998), Düring-Olsen et al. (1999), Iraqui et al. (1999), Regenberg et al. (1999), Andréasson et al. (2004), Schreve and Garrett (2004) Sáenz et al. (2014), and Ruiz et al. (2021)
Can1	YEL063C	His, Arg (10–20 μM), Lys (150–250 μM), Orn	Grenson et al. (1966), Regenberg et al. (1999), Ghaddar et al. (2014a), and Ruiz et al. (2021)
Gap1	YKR039W	All natural amino acids, Cit, Orn, Leu (20 μM)	Grenson et al. (1970), Regenberg et al. (1999), Schreve and Garrett (2004), and Risinger et al. (2006)
Lyp1	YNL268W	Met, Lys (10–25 μM)	Grenson et al. (1966), Sychrová et al. (1993), Sychrova and Chevallier (1993), Regenberg et al. (1999), Ghaddar et al. (2014a), and Ruiz et al. (2021)
Mup1	YGR055W	Met, Cys	Isnard et al. (1996) and Kosugi et al. (2001)

Values in brackets indicate reported Michaelis constants (Km).

### Transporters’ effect on intracellular amino acid concentrations

Amino acid homeostasis is tightly regulated in living organisms (Magasanik and Kaiser, 2002; Ljungdahl, 2009; Ljungdahl and Daignan-Fornier, 2012). Therefore, we first aimed to evaluate the effect of the deletion or overexpression of the five transporters on the total intracellular amino acid concentrations. In parallel, to test the viability of the transporter mutant strains, a growth assay for 72 h was completed.

Hierarchical cluster analysis revealed two distinct groups of transporter mutants based on their average amino acid concentrations. One cluster consisted of transporter overexpression and another of deletion mutant strains (Figure 2).

The cluster of strains overexpressing Lyp1, Gap1, and Can1 had minor negative to no effect on the total amino acid concentrations. In contrast, the strain overexpressing the broad-range Agp1 transporter had the highest average intracellular concentration of the 13 analyzed amino acids compared to the wild-type strain. Among others, a substantial increase in concentrations was observed for Asn-L (0.67 ± 0.14 mM), Phe-L (1.32 ± 0.26 mM), Glu-L (84.71 ± 10.34 mM), Trp-L (0.19 ± 0.04 mM) and Tyr (4.14 ± 0.63 mM) (Figure 2). Intriguingly, overexpression of the narrow-range Mup1 permease led to similar results with increased concentrations for 11 amino acids. Therefore, it was unsurprising that Mup1 and Agp1 overexpression strains clustered together based on their amino acid profiles.

Deletion of the lysine permease Lyp1 had almost no effect on the amino acid profile of the strain, except for a slight increase in Met-L (1.17 fold), Phe-L (1.26 fold), and Trp (1.27 fold) and a decrease in His-L (0.2 fold) concentrations. Therefore, the Lyp1 mutant strain clustered together with the background strain. In comparison, deletion of the Agp1 permease led to a significant decrease in the intracellular concentrations of Asn-L, Gln-L, Glu-L, Thr-L, and Tyr-L. A significant reduction in Asn-L, Gln-L, His-L, and Thr-L concentrations was also observed for the Gap1 deletion mutant. Except for His-L, a similar reduction in the three amino acid concentrations was observed upon deletion of the Can1 transporter. The amino acid profile of the Mup1 deletion mutant placed it furthest away from the rest of the transporter deletion mutants. Despite having a minor effect on 11 of the amino acids, deletion of Mup1 had a positive effect on Orn (1.57 fold increase) and a significant increase in Ser-L (0.41 ± 0.03 mM) concentrations (Figure 2).

Except for Agp1, the observed fluctuations in the amino acid pools of the yeast transporter mutants did not affect the cells’ growth profiles (Supplementary Figure S1). Overexpression of Agp1 led to retarded growth of the yeast cells with a maximum OD_600_ of 2.4 after 31 h. In contrast, the corresponding deletion mutant showed a slight growth improvement (~1.2 fold) compared to the background strain.

These findings suggested that the five transporters, to varying degrees, contribute to yeast’s amino acid homeostasis. Since the determined amino acid concentrations are based on total intracellular amounts, it was not possible to account for whether the observed differences were due to transport events or the deregulation of the amino acid biosynthetic pathways. Hence, it was necessary to differentiate between *de novo* synthesized and exogenously supplied amino acids to investigate the reason for the observed differences.

### Amino acid transport assay in *Saccharomyces cerevisiae*

To investigate further to what extent uptake and excretion contributed to the observed differences in the amino acid concentrations, we continued with isotopic labeling of the yeast endometabolites followed by a transport assay.

Principal component analysis (PCA) models of both the intracellular and the extracellular extracts revealed distinguishable differences (Figure 3). Furthermore, the distribution on PC1 and PC2 score plot showed the relative grouping of the biological replicates for most yeast transporter mutants.

The analysis of intracellular extracts found that all overexpression strains, except Agp1, had minimal changes in amino acid profiles (Figure 3A). Agp1 overexpression resulted in a different transport profile due to a positive correlation with Arg-L and Citr-L. Gap1 deletion mutant had the highest amino acid dissimilarity and was defined by seven amino acids. Can1 deletion mutant negatively correlated to Arg-L, confirming its role as an arginine transporter. The Mup1 deletion mutant showed an altered amino acid uptake profile for two of the biological replicates.

PCA analysis of extracellular extracts revealed clear differences between transporter overexpression and deletion mutants compared to intracellular data (Figure 3B). Agp1, Can1, Gap1, and Mup1 deletions showed a positive correlation with Arg-L, Gln-L, Ser-L, Tyr-L, and Met-L quadrant, implying reduced uptake and possible excretion of these amino acids. Lyp1 deletion had minimal impact on the yeast mutant amino acid profile, while the Gap1 overexpression strain grouped with the reference strain. The rest of the overexpression strains were distributed toward the plot’s periphery. Can1, Mup1, and Lyp1 overexpression led to negatively correlated extracellular amino acid profiles with the respective deletion variants, as anticipated. Agp1 overexpression stood out, displaying a unique scatter pattern with covaried Asn-L, Phe-L, Thr-L, and Glu-L, hinting at potential amino acid excretion involvement.

The contribution of amino acids to the PCA model and their correlation with transport was revealed by their coefficients and position on the loading plot (Supplementary Table S5 and Figures 3C,D). The intracellular extracts showed that Arg-L had the strongest impact on the second principal component, and Thr-L, Met-L, Phe-L, and Tyr-L had a positive correlation, indicating interdependent transport. Extracellular amino acids fell into two independent groups, with Phe-L, Thr-L, Asn-L, and Glu-L in one group and Arg-L, Gln-L, Ser-L, Tyr-L, and Met-L in another. Cit-L and Asp-L were transported differently from other amino acids. Arg-L had the strongest impact on PC2.

Altogether, PCA analyses of intra- and extracellular metabolite extracts revealed distinct correlations among the five transporter deletion and overexpression mutants. These correlations reflected the substrate preference for each of the five amino acid carriers.

To understand the distinct transport patterns observed in PCA analysis, we examined relative isotopologue ratios in intra- and extracellular metabolite extracts for each amino acid. For quantifying transport levels, we compared ^13^C and ^12^C isotope ratios of transporter deletion mutants and overexpressing strains to the wild-type under the same conditions (Figure 4).

Agp1 overexpression led to significant intracellular accumulation of Arg-L, Glu-L, Thr-L, and Met-L. Notably, Agp1 overexpression also resulted in increased extracellular Glu-L and Thr-L levels. In addition, the Agp1 deletion mutant showed high intracellular Phe-L, Tyr-L, and Met-L, while extracellular amino acid levels remained unaffected.

As anticipated, Can1 deletion reduced intracellular Arg-L levels and increased them in the extracellular fraction. Overexpression of Can1 had the inverse effect on the extracellular Arg-L levels and no significant change in the amino acid endometabolite content. Unexpectedly, deleting Can1 resulted in elevated intracellular levels of Asn-L, Asp-L, Glu-L, Phe-L, Thr-L, Tyr-L, and Met-L.

Conversely, yeast strains overexpressing Can1 exhibited reduced extracellular content of Glu-L, Phe-L, Ser-L, Thr-L, and Tyr-L, along with a notable decrease in Phe-L, Tyr-L, and Met-L in the endometabolite analysis. Although Lyp1 and Mup1 are narrow-range transporters, the Lyp1 deletion mutant had surprisingly increased Phe-L, Tyr-L, and Met-L intracellularly and elevated Thr-L and Tyr-L extracellularly. Lyp1 overexpression decreased extracellular Glu-L, Phe-L, Thr-L, and Tyr-L and had no effect on the amino acids intracellular content. For the Mup1 overexpression strain, decreased extracellular Arg-L, Asn-L, Phe-L, Ser-L, Thr-L, and Tyr-L levels were observed. Overexpression of Mup1 also decreased intracellular Phe-L and Tyr-L content. Moreover, Mup1 deletion raised extracellular Arg-L, Ser-L, and Gln-L while inversely affecting Phe-L and Thr-L outside the cells. Additionally, Mup1 deletion led to higher intracellular Asn-L, Phe-L, Thr-L, Tyr-L, and Met-L. Deletion of Gap1, a broad-range amino acid permease, had a substantial impact on nearly all amino acid levels. Intriguingly, Gap1 deletion significantly increased intracellular levels of Asn-L, Asp-L, Glu-L, Phe-L, Thr-L, Tyr-L, and Met-L. The notable decrease in Glu-L and Phe-L observed in exometabolite analysis was consistent with the intracellular findings. However, Gap1 deletion had the opposite effect on Arg-L, causing decreased intracellular levels and increased extracellular content. Gap1 overexpression also affected amino acid transport, albeit to a lesser extent than the deletion mutant variant. Gap1 overexpression strain showed elevated Phe-L, Thr-L, Tyr-L, and Met-L in the yeast endometabolite extracts, while Arg-L levels were reduced. Extracellular extracts of the Gap1 overexpression strain revealed increased Arg-L but significantly lower amounts of Phe-L, Thr-L, and Tyr-L.

Overall, the five amino acid transporters exhibited distinct uptake and excretion patterns for the 11 amino acids. However, due to *S. cerevisiae* amino acid transporters’ promiscuity and overlapping functions, the observed transport patterns needed further validation. Therefore, we next set up an uptake assay in *Xenopus* oocytes expressing the single amino acids transporters. In this way, we aimed to omit background transport activity and investigate the single transporter uptake profile.

### Validation of amino acid transport in *Xenopus* oocytes

*Xenopus* oocytes have proven to be a valuable heterologous system for characterizing transporter proteins from a wide range of organisms (Miller and Zhou, 2000; Wagner et al., 2000). Motivated by their well-described low background transport activity, we employed this system to express the five *S. cerevisiae* amino acid carriers. To achieve this, we injected oocytes with specific cRNA encoding each of the five *S. cerevisiae* transporters, supplemented with cRNA encoding free GFP to control for potential endogenous transporter-mediated amino acid uptake. The transport assay involved incubating the injected oocytes with a uniformly labeled ^13^C yeast extract derived from *P. pastoris*, followed by comprehensive LC–MS/MS analysis of the extracted metabolite contents (Figure 5A).

From the intracellular extracts of the injected oocytes, we successfully detected ten ^13^C-labeled amino acids, namely Arg-L, Asn-L, Asp-L, Cit, Cys-L, Gln-L, Glu-L, His-L, Met-L, and Thr-L (Figure 5B). However, due to the detection of fewer than three biological replicates, Asp-L and Glu-L were excluded from further data analysis (Supplementary Figure S4).

In the control group of oocytes injected with GFP, we observed uptake of ^13^C-labeled amino acids solely for Gln-L (1608.64 ± 501.22 AU) and Citr (396.49 ± 53.23 AU). The remaining six amino acids in the GFP control extract were below the detection limit of our instrument. Consequently, we attributed the increased intracellular levels of the detected amino acids, excluding Gln-L and Citr, to uptake by the amino acid carriers.

Expression of Agp1 led to the notable accumulation of Arg-L, consistent with the uptake assay performed in *S. cerevisiae*. As expected, oocytes injected with Can1 exhibited increased levels of Arg-L. Moreover, Can1 facilitated the import of Asn-L and Cys-L. Similar uptake profiles were observed for Lyp1, resulting in the intracellular accumulation of Arg-L and Asn-L but not Cys-L.

Consistent with previously reported substrate specificities, Mup1 mediated the uptake of Met-L. Interestingly, Mup1 also facilitated the accumulation of Asn-L, His-L, and Thr-L in the oocyte cells. Although we observed Citr-L uptake by Mup1, it did not reach statistical significance compared to the control strain.

Collectively, our results from the *Xenopus* oocyte uptake assay demonstrated uptake profiles by the five transporters that partially aligned with our findings in the yeast transport assay (Table 2). However, two transporters, Gap1 and Mup1, exhibited altered uptake profiles between the two assays.

**Table 2:** Summary table of amino acid transport assays in *S. cerevisiae* and *Xenopus* oocytes.^a^

		**Arg-L**		**Asn-L**		**Asp-L**		**Gln-L**		**Glu-L**		**Met-L**		**Phe-L**		**Ser-L**		**Thr-L**		**Tyr-L**		**Cys-L**		**His-L**	
	Cell line	IN	EX	IN	EX	IN	EX	IN	EX	IN	EX	IN	EX	IN	EX	IN	EX	IN	EX	IN	EX				
**Agp1**	OE	+								+	+	+							+	+					
	Deletion											+		+				+							
**Can1**	OE		−								−	−		−	−		−	−	−	−	−				
	Deletion	−	+	+		+				+		+		+	−		+	+	−	+					
**Lyp1**	OE										−				−				−	−	−				
	Deletion											+		+					+		+				
**Gap1**	OE	−	+			+						+		+	−			+	−	+	−				
	Deletion	−	+	+		+				+	−	+		+	−		+	+							
**Mup1**	OE		−		−									−	−		−		−	−	−				
	Deletion		+	+					+			+		+	−		+	+	−	+					

^a^Amino acid levels for *S. cerevisiae* transport assay: OE, overexpression; IN, intracellular; EX, extracellular; +, increase in levels; −, decrease in levels; thick border line, reported substrate. Grey fill denotes the uptake of amino acids in *Xenopus* oocytes.

## Discussion

Methodologies for studying transporter proteins have been heavily dependent on radioactively labeled substrates or toxic analogs. The approach presented here shows proof of principle for studying the transport of small molecules using the ^13^C labeled metabolites measured by LC–MS/MS (Figure 1). As a case study, we selected five well-described *S. cerevisiae* amino acid transporters with varying substrate ranges.

A comprehensive analysis of the global impact on amino acid levels upon transporter overexpression or deletion (Figure 2) showed results partially consistent with previously reported data. Notably, overexpression of Mup1 elevated intracellular levels of all analyzed amino acids except for Asn-L and Thr-L. Interestingly, viability assessment through time-course growth experiments revealed that only Agp1 overexpressing cells exhibited retarded growth, reaching an OD_600_ = 2.37 after 31 h (Supplementary Figure S1). This growth phenotype of the Agp1 overexpression strain may be attributed to the inhibitory effect of increased uptake of free amino acids (Ruiz et al., 2021).

It is essential to recognize that the total intracellular amino acid concentrations observed in this analysis cannot be solely attributed to the uptake of external amino acids, as they cannot be distinguished from amino acids synthesized *de novo*. Therefore, the observed changes may result from both transport processes and the regulation of amino acid biosynthetic pathways. These regulatory mechanisms, such as the general amino acid control (GAAC) system, can respond to the availability of external amino acids (Ljungdahl and Daignan-Fornier, 2012).

To elucidate the contributions of import and export to the observed differences in intracellular amino acid levels among the yeast transporter mutants, we conducted two transport assays using ^13^C-labeled metabolites. Our *S. cerevisiae* transport assay depends on nearly full isotope metabolite labeling. Therefore, to achieve ^13^C metabolite replacement, cells were cultured in uniformly labeled ^13^C-glucose for 21 h. Previously reported shorter cultivation times of 4 h (Theobald et al., 1997; Visser et al., 2002) conflicted with more recent data (Mashego et al., 2004), which showed that only 80% of glycolytic metabolites were ^13^C replaced during short cultivation periods. Thus, we followed Mashego et al. (2004) recommendations to increase the cultivation time to accommodate a larger replacement of the glycolytic metabolites exceeding 80%, primarily to reduce the standard deviation in the measured changes of amino acids levels caused by transport activity.

The *S. cerevisiae* assay revealed distinct amino acid transport profiles in the yeast single transporter mutants (Figure 3), resulting in significant changes in individual amino acid levels (Figure 4 and Supplementary Figure S3). We observed both expected results and unexpected alterations in amino acid levels that did not conform to established substrate specificities (Table 2). We speculate that multilevel regulation of amino acid transporters in *S. cerevisiae* could account for these unexpected results. The interplay of amino acid transporter regulation through trafficking (Lin et al., 2008; Nikko and Pelham, 2009; Hatakeyama et al., 2010; Ghaddar et al., 2014b; Gournas et al., 2018) and domain partitioning (Bianchi et al., 2018; Busto et al., 2018; Gournas et al., 2018) could potentially account for the unexpected transport profiles observed in our yeast transporter mutant strains.

An intriguing observation in our yeast transport assay was the absence of Glu-L transport in the Mup1 overexpressing strain (Figure 4), contrasting with the significant intracellular accumulation of this amino acid indicated by the global amino acid analysis (Figure 2). While we assessed total intracellular amino acid concentrations in the transporter mutants, we did not account for changes in extracellular levels. Additionally, our approach, representing transporter activity in *S. cerevisiae* through isotopologue ratios, does not consider intracellular amino acid levels but rather serves as a relative measure compared to the background strain. We hypothesize that the divergent outcomes between the two assays may result from potential cross-talk between the Ssy1-Ptr3-Ssy5 (SPS), GAAC, and nitrogen catabolite repression (NCR) systems (reviewed in Bianchi et al., 2019). This interplay could redirect metabolic flux toward specific amino acids, even as these same amino acids are concurrently imported by the constitutively expressed transporter. In such a scenario, exemplified by Mup1 and Glu-L, intracellular amino acid concentrations increase significantly, while the ratio between imported and *de novo* synthesized amino acids remains comparable to the background strain.

The substantial accumulation of amino acids in various single transporter deletion strains observed in this study can also be attributed to the promiscuity, overlapping specificities, and the abundance of amino acid transporters in the yeast plasma membrane (Bianchi et al., 2019). Future application of our method should address the challenges associated with transporter redundancy and overlapping functions in *S. cerevisiae*, necessitating careful selection of suitable background transporter deletion strains.

The surprising substrate specificities of the five amino acid transporters could also be due to previous studies focusing on a limited panel of substrates and lower concentrations. Notably, Ruiz et al. (2021) examined amino acid growth inhibition using a mixture of 20 amino acids at 10 times higher concentrations than our study. Interestingly, their results revealed broader substrate specificity for several amino acid transporters compared to previous reports. In this respect, an inherent advantage of our approach lies in its ability to simultaneously screen multiple metabolites against a candidate transporter. Unlike fluorescent-based methods, which are constrained by the number of available fluorescent molecules and scintillation counting techniques, primarily limited to three main radioactive isotopes (^3^H, ^14^C, and ^32^P), our targeted approach offers the capability to analyze over 100 metabolites from various classes concurrently. By utilizing commercially available ^13^C-labeled mixes, such as canonical amino acids, tricarboxylic acid cycle intermediates, and sugars, it would become feasible to initially narrow down the library of screened metabolites to specific target groups. Subsequently, the method can be expanded to include the complete metabolite mix, similar to the ^13^C yeast extract from *Pichia pastoris* utilized in this study. This feature provides a substantial advantage, as it exposes the transporter proteins of interest to a wide array of substrates, thus closely mimicking their native physiological environment. Moreover, from a time-efficiency perspective, our method generates information-rich data for hundreds of metabolites per transporter within a single week of analysis, whereas classical transport assays necessitate multiple rounds of screening against potential metabolites, often spanning several months.

The unexpected results from the *S. cerevisiae* uptake assay led us to further evaluate our method by heterologously expressing the five amino acid transporters in *Xenopus* oocytes (Figure 5). With this, we aimed to minimize the background transport activity and regulatory mechanisms otherwise found in *S. cerevisiae*.

To account for any background uptake, we employed GFP-injected oocyte cells as a control. The GFP-positive oocytes displayed detectable levels of Citr and Gln-L accumulation. This result was not unexpected since it has been previously shown that *Xenopus* oocytes have several endogenous Na^+^ − dependent amino acid uptake systems (Van Winkle, 1993; Taylor et al., 1996; Sobczak et al., 2010). Our observations of *S. cerevisiae* transporters expressed in *Xenopus* oocytes revealed uptake profiles that partly aligned with previous research and our *S. cerevisiae* assay (Table 2).

All *S. cerevisiae* transporters expressed in the oocyte cells displayed uptake activity except for Gap1, which did not facilitate any uptake. One possible explanation for Gap1’s lack of uptake activity is the potential issue with its expression or integration into the oocyte’s plasma membrane. To test the latter hypothesis, one would perform a western-blot analysis of total oocyte membrane proteins and appropriately tagged (e.g., FLAG, HIS, GFP) Gap1. However, since our objective was to design a strategy that can be applied for high-throughput screening of transporters and membrane preparation and immunoblotting are notoriously time-consuming procedures, we did not include them in our approach. Yet, we did monitor the capability of the oocyte cells to express foreign mRNA by selecting cells co-expressing GFP. Future improvements could explore the utilization of oocyte-adapted high-throughput microscopy with fluorescently labeled yeast transporters in *Xenopus* oocytes. This approach would facilitate rapid screening of transporter proteins localized in the plasma membrane of *Xenopus* oocyte cells prior to subjection to transport assay.

The disparities observed between the *S. cerevisiae* and *Xenopus* oocyte transport assays may be attributed to variations in amino acid concentrations in the amino acid mix and the ^13^C uniformly labeled yeast extract from *P. pastoris*. In the *S. cerevisiae* transport assay, we utilized concentrations of amino acids within the range of 10–300 μM, as commonly employed in synthetic media preparation and consistent with prior literature (Regenberg et al., 1999; Schreve and Garrett, 2004). Conversely, the ^13^C uniformly labeled yeast extract from *P. pastoris* contains amino acid concentrations approximately tenfold lower. Moreover, the yeast extract comprises a complex mixture of hundreds of metabolites, more closely mirroring the natural conditions in which the transporters operate. This increased metabolite diversity in the yeast extract intensifies substrate competition, potentially leading to transporter inhibition.

Our method holds a distinct advantage in detecting the transport of *S. cerevisiae* native metabolites. While our focus has been on showcasing the transport of amino acids, it is important to note that our approach can be extended to encompass other native metabolite classes, including organic acids, bases, sugars, carbohydrates, and nucleosides (McCloskey et al., 2015). One current limitation of our method lies in the number of metabolites it can target within our targeted LC–MS/MS detection framework. To further expand the scope and accommodate a plethora of detected metabolites, an untargeted LC–MS/MS approach can be implemented, followed by metabolite annotation and the utilization of reference standards. This approach has the potential to extend the coverage to thousands of metabolites, as demonstrated in recent studies investigating the differential uptake profiles of mammalian cell lines when incubated with blood serum (Wright Muelas et al., 2020). Another label-free strategy to study transport proteins applied to mammalian cells involves the use of matrix-assisted laser desorption/ionization (MALDI) coupled with a time-of-flight (TOF) mass analyzer (Unger et al., 2021). While this technology offers good sensitivity and relatively high-throughput screening potential in comparison to our method, the challenges in matrix selection for specific metabolite groups remain a significant limitation.

A notable challenge in our LC–MS/MS approach pertains to sample preparation, involving metabolite extraction with either methanol or ethanol, followed by subsequent evaporation. It is important to acknowledge that no single metabolite chemical extraction method currently exists that ensures the complete recovery of all metabolite classes (Villas-Bôas et al., 2005). Therefore, future optimization of our method should consider the specific metabolite class of interest and select the extraction method accordingly. For instance, while boiling ethanol has proven to be an effective extraction reagent for amino acids and organic acids and relatively suitable for fatty acids, it is not appropriate for other classes, such as nucleotides, sugars, and sugar phosphates (Villas-Bôas et al., 2005). This underscores the importance of a tailored approach to extraction in our methodology.

In our current investigation, we primarily focused on *S. cerevisiae* transporters reported to function at the plasma membrane. However, it is important to acknowledge that many eukaryotic transporters are involved in the transport of metabolites between the intracellular compartments of the cell. Studying these intracellular transporters poses challenges, as *in vivo* transport assays in *S. cerevisiae* typically rely on externally provided metabolites. An exceptional advantage of our method is the ability to heterologously express *S. cerevisiae* intracellular transporters in *Xenopus* oocytes, facilitating the study of their activity. This is achieved by fusing a leader peptide to the transporter of interest, enabling its targeting to the oocyte’s plasma membrane, as previously demonstrated for the mitochondrial transporter Ctp1 (Darbani et al., 2019).

Furthermore, future optimizations of our method may involve the investigation of exporters, for which currently, only limited methods are available. Our current method allowed the detection of amino acid exporters, as we observed the excretion of 13 amino acids from the background yeast strain after incubation with a ^13^C labeled metabolite yeast extract (Supplementary Figure S2). Yet, further optimization of the method could be achieved by injecting ^13^C-labeled metabolite mixes of interest into the oocyte cells. Subsequently, analyzing the decrease in intracellular levels or accumulation in extracellular levels of the metabolite could be performed, as demonstrated in a recent work with the *E. coli* Lys-L transporter MglE (Malla et al., 2022).

This work introduces a promising method for identifying metabolite transporters in *S. cerevisiae,* employing LC–MS/MS analysis and isotope labeling. Our method enables the simultaneous screening of multiple transporters against selected metabolite libraries. We applied this approach to study five *S. cerevisiae* amino acid transporters in two independent transport assays. The first assay revealed reproducible differences in the uptake or export of 11 amino acids between the transporter mutant cell lines and the yeast background strain. These results unveiled broader substrate specificity for some transporters compared to previous reports. We successfully extended the method to *Xenopus* oocytes heterologously expressing the five *S. cerevisiae* amino acid transporters, demonstrating partial complementation. Although the assay results varied from initial expectations based on the literature, we tentatively propose broader substrate specificity for Mup1. Moreover, our data unequivocally indicates the uptake of Arg-L by Agp1, a previously unreported substrate. Given the overwhelming number of uncharacterized *S. cerevisiae* transporters, we firmly believe that the utilization of our method will significantly contribute to their deorphanization and establish a robust foundation for subsequent functional analysis.

## Data availability statement

The original contributions presented in the study are included in the article/Supplementary material, further inquiries can be directed to the corresponding author.

## Author contributions

LS: Conceptualization, Data curation, Investigation, Methodology, Software, Validation, Visualization, Writing – original draft, Writing – review & editing. IM-H: Investigation, Methodology, Supervision, Validation, Writing – review & editing. PL: Data curation, Investigation, Writing – review & editing. CR: Investigation, Software, Writing – review & editing. IB: Conceptualization, Funding acquisition, Project administration, Resources, Supervision, Writing – review & editing.
