# Supplementary material for: Screening of Saccharomyces cerevisiae metabolite transporters by 13C isotope substrate labeling
Source: Front Microbiol. 2023 Nov 27;14:1286597. doi: 10.3389/fmicb.2023.1286597 (PMC10729909; doi:10.3389/fmicb.2023.1286597)
Supplement: Supplementary file 1 [file Data_Sheet_1.PDF]

Supplementary Material

1 Supplementary Figures

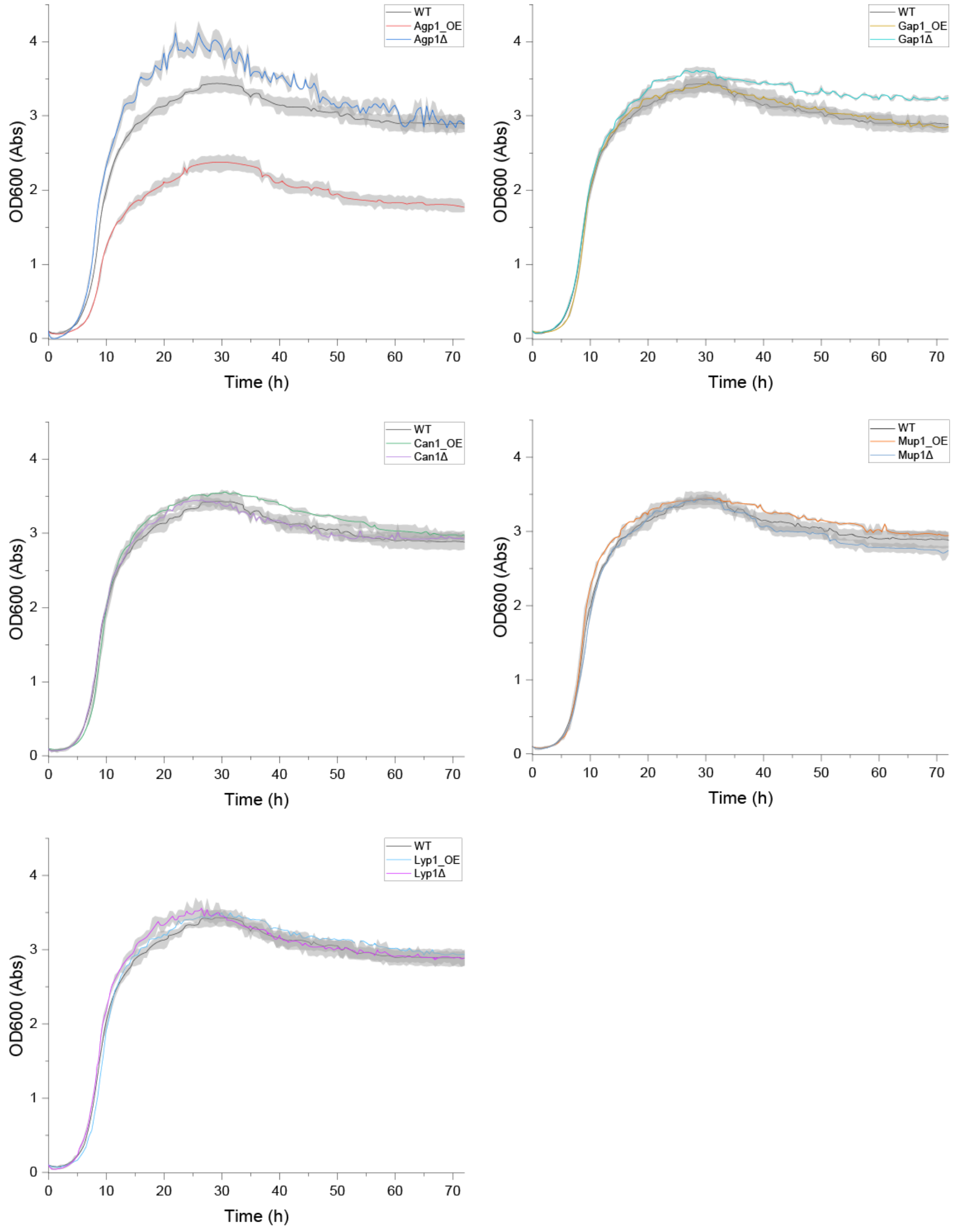

Supplementary Figure 1. Growth profile of amino acid transporter mutants. Yeast mutant strains with deleted or overexpressing Agp1, Can1, Gap1, Lyp1 or Mup1 amino acid transporters were grown in minimal medium with unlabelled glucose for 72 h. Measurements were acquired every 50 min. Results are the average of two biological replicates with SD±. OE, overexpression strain; Δ, deletion mutant; WT, wild-type strain.

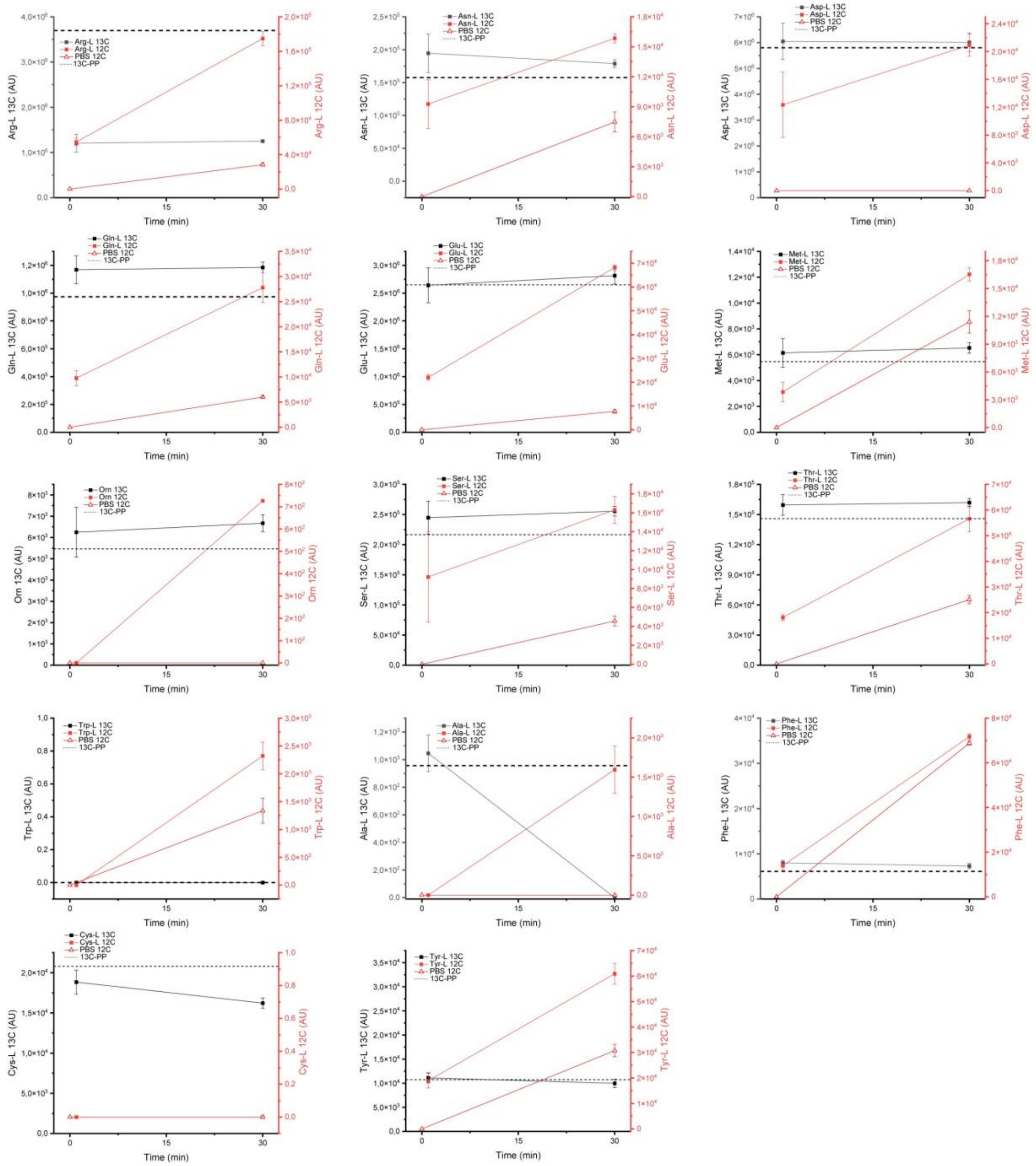

Supplementary Figure 2. Time-course transport of amino acids. Wild-type yeast cells were grown in a minimal medium with natural glucose and subsequently incubated with ^{13}C uniformly labeled metabolite yeast extract or in PBS without amino acids. Extracellular ^{12}C- (filled red square) and ^{13}C- (filled black square) amino acids were then analyzed after 1 and 30 min of incubation with the ^{13}C metabolite yeast extract. Extracellular amino acids from cells incubated with PBS (open red triangle) were analyzed directly (0 min) and after 30 min. Black dashed line indicates the reference values of the amino acids in the ^{13}C yeast extract. Results are the average of three biological replicates with SD±.

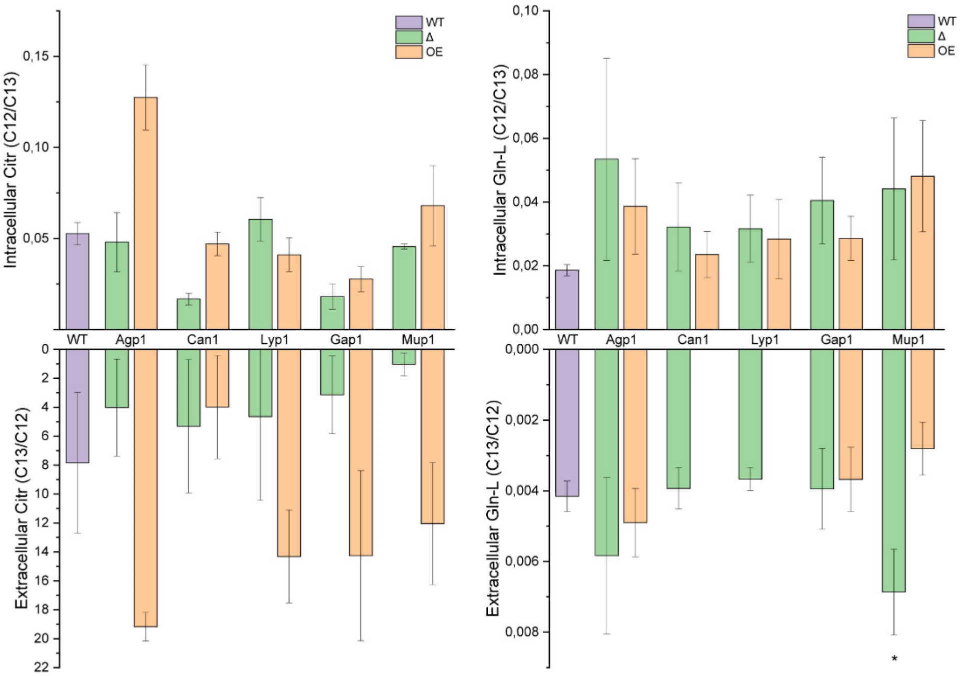

Supplementary Figure 3. Transport of Citr-L and Gln-L in *S. cerevisiae*. Intra- and extracellular analysis of extracted Citr-L and Gln-L. Yeast mutant strains with deleted or overexpressing Agp1, Can1, Gap1, Lyp1 or Mup1 amino acid transporters were grown in medium with ^{13}C-glucose and subsequently incubated with unlabeled amino acid mix. The graphs represent the ratios of the peak areas for ^{12}C and ^{13}C single amino acids ($^{12}C/^{13}C$ ratio for intracellular and $^{13}C/^{12}C$ for extracellular transport). Results are the average of three biological replicates with SD±, except for Citr-L, which is the result of two biological replicates. Extracellular Gln-L for the Can1 and Lyp1 overexpression strains was not detected. Student t-test indicates a statistically significant difference compared to the wild-type strain. (*p-value < 0.05). OE, overexpression strain; Δ, deletion mutant; WT, wild-type strain.

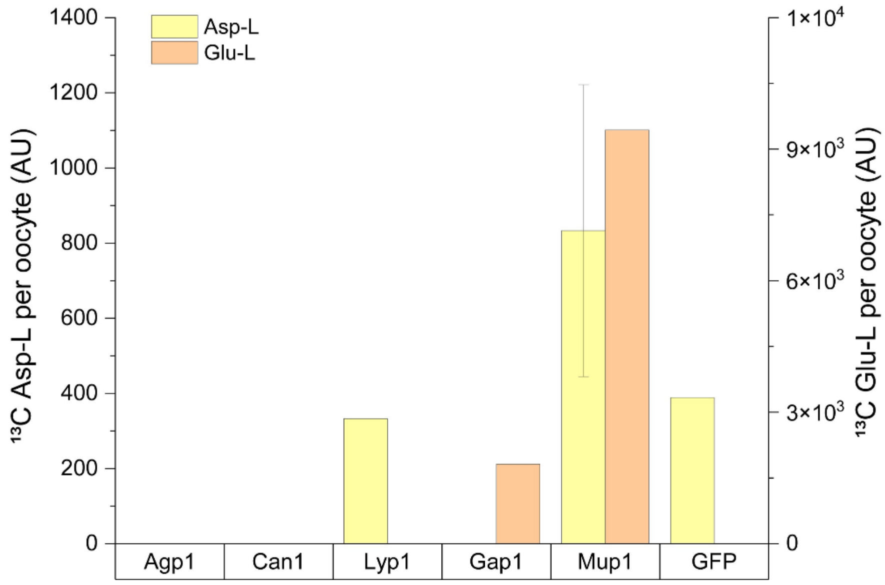

Supplementary Figure 4. Uptake of Asp-L and Glu-L in *Xenopus* oocytes. Analysis of ^{13}C intracellular amino acid levels from *Xenopus* oocytes expressing single *S.cerevisiae* transporters or GFP. The right y-axis indicates the intracellular levels of ^{13}C Gln-L, and the left y-axis of Asp-L. Results are obtained from one biological replicate (each replicate consists of 3-4 oocytes) except for Asp-L levels in Mup1 expressing cells, which is the average result of two replicates with SD±.

2 Supplementary tables

Supplementary Table 1. List of strains used in this study.

Strain	Genotype	Plasmid	Reference
BY4741	MATα; *his3Δ1; leu2Δ0; met15Δ0; ura3Δ0*		Open Biosystems
3432	BY4741 carrying KanMX::AGP1		Open Biosystems
305	BY4741 carrying KanMX::CAN1		Open Biosystems
7385	BY4741 carrying KanMX::LYP1		Open Biosystems
7050	BY4741 carrying KanMX::GAP1		Open Biosystems
4685	BY4741 carrying KanMX::MUP1		Open Biosystems
ST11548	BY4741	pCfB10960 for overexpression of AGP1	This study
ST11549	BY4741	pCfB10961 for overexpression of CAN1	This study
ST11550	BY4741	pCfB10962 for overexpression of LYP1	This study
ST11551	BY4741	pCfB10963 for overexpression of GAP1	This study
ST11552	BY4741	pCfB10964 for overexpression of MUP1	This study

Supplementary Table 2. Oligonucleotide primers used in this study.

Name	Sequence (5' to 3')	Orientation	Description
PR-29392	CGTGCGUGCACACACCATAGCTTCAAAATG	→	Amplification of pTEF1 promoter
PR-29393	ATGACAGAUTTGTAATTAAAACTTAGATTAGATTG	←	
PR-29394	ATCTGTCAUATGTCGTCGTCGAAGTCTCTATACGAACT	→	Amplification of AGP1 from gDNA
PR-29395	CACGCGAUTTAACACCAGAAGGCAACGACCCT	←	
PR-29396	ATCTGTCAUATGACAAATTCAAAAGAAGACGCCGACA	→	Amplification of CAN1 from gDNA
PR-29397	CACGCGAUCTATGCTACAACATTCCAAAATTTGTCCCAAAAAGTCT	←	
PR-29398	ATCTGTCAUATGGGCAGGTTTAGTAACATAATAACGTCCAATAAATGG	→	Amplification of LYP1 from gDNA
PR-29399	CACGCGAUCTATGCAACAGCAGCCCAGAATTTCTCC	←	
PR-29400	ATCTGTCAUATGAGTAATACTTCTTCGTACGAGAAGAATAATCCAGATAATCTGAAAC	→	Amplification of GAP1 from gDNA
PR-29401	CACGCGAUTTAACACCAGAAATTCCAGATTCTATACCATCTTGGC	←	
PR-29402	ATCTGTCAUATGTCGGAAGGAAGAACGTTTCTGTCAC	→	Amplification of MUP1 from gDNA
PR-29403	CACGCGAUTTACAGCGATTTTTCTTGTTCACTTTTGTAATGTTCGAT	←	
PR-31946	GGCTTAAUATGTCGTCGTCGAAGTCTCTATACG	→	Cloning of AGP1 into pUSER016 vector
PR-31947	GGTTTAAUTTAACACCAGAAGGCAACGACC	←	

PR-31948	GGCTTAAUATGACAAATTCAAAAG AAGACGCCGAC	→	Cloning of CAN1 into pUSER016 vector
PR-31949	GGTTTAAUCTATGCTACAACATTCC AAAATTTGTCCCAAA	←	
PR-31950	GGCTTAAUATGGGCAGGTTTAGTA ACATAATAACGTCC	→	Cloning of LYP1 into pUSER016 vector
PR-31951	GGTTTAAUCTATGCAACAGCAGCC CAGAAT	←	
PR-31952	GGCTTAAUATGAGTAATACTTCTTC GTACGAGAAGAATAATCCAG	→	Cloning of GAP1 into pUSER016 vector
PR-31953	GGTTTAAUTTAACACCAGAAATTCC AGATTCTATACCATCT	←	
PR-31954	GGCTTAAUATGTCGGAAGGAAGAA CGTTTCTG	→	Cloning of MUP1 into pUSER016 vector
PR-31955	GGTTTAAUTTACAGCGATTTTTCTT GTTCACTTTTGTAATG	←	

Supplementary Table 3. Plasmids used in this study.

Name	Description	Reference
pCfB2225	Template, EasyClone integration vector pXII-2-loxP-KanMXsyn	(Jessop-Fabre *et al.*, 2016)
pCfB10960	Overexpression of AGP1 under pTEF promoter	This study
pCfB10961	Overexpression of CAN1 under pTEF promoter	This study
pCfB10962	Overexpression of LYP1 under pTEF promoter	This study
pCfB10963	Overexpression of GAP1 under pTEF promoter	This study
pCfB10964	Overexpression of MUP1 under pTEF promoter	This study
pUSER016	USER-compatible *Xenopus* expression vector	(Nour-Eldin *et al.*, 2006)
pCfB11871	pUSER016 carrying MUP1 gene	This study
pCfB11870	pUSER016 carrying GAP1 gene	This study
pCfB11869	pUSER016 carrying LYP1 gene	This study
pCfB11868	pUSER016 carrying CAN1 gene	This study
pCfB11867	pUSER016 carrying AGP1 gene	This study
pCfB8156	pUSER016 carrying GFP gene	(Møller-Hansen I. *et al*. unpublished)

Supplementary Table 4. Intracellular amino acid concentrations (mM).

	Arg-L	Asn-L	Citr-L	Gln-L	Glu-L	His-L	Met-L	Orn	Phe-L	Ser-L	Thr-L	Trp-L*	Tyr-L
Agp1_OE	19,66±2,98	0,67±0,14	0,21±0,03	1,28±0,08	84,72±10,34	-	0,46±0,03	9,29±1,61	1,32±0,26	0,36±0,07	6,73±0,95	189,66±40,32	4,14±0,63
Can1_OE	10,88±0,96	0,41±0,1	0,13±0,02	0,56±0,14	15,89±1,61	8,19±4,68	0,21±0,01	3,00±0,61	0,58±0,07	0,29±0,07	4,73±0,90	48,11±2,14	1,39±0,23
Lyp1_OE	10,18±0,93	0,48±0,04	0,13±0,02	0,80±0,05	15,13±1,93	10,00±1,90	0,15±0,02	3,04±0,58	0,56±0,08	0,31±0,05	4,95±0,64	45,24±6,36	1,39±0,17
Gap1_OE	9,97±1,03	0,38±0,08	0,12±0,02	0,56±0,2	15,64±0,21	9,35±1,18	0,19±0,03	3,15±0,50	0,51±0,09	0,25±0,08	4,36±0,87	43,63±8,43	1,39±0,21
Mup1_OE	18,09±2,42	0,41±0,09	0,27±0,03	1,30±0,36	**93,18±6,19**	21,65±2,48	**0,51±0,06**	5,59±0,90	0,88±0,18	0,38±0,11	3,58±0,74	**196,98±24,37**	**3,87±0,52**
Agp1Δ	10,03±0,75	**0,19±0,02**	0,14±0,02	**0,41±0,01**	**9,13±0,27**	13,82±0,52	0,19±0,02	3,34±0,35	0,31±0,02	0,27±0,04	**2,11±0,19**	42,33±2,73	**0,90±0,06**
Can1Δ	12,34±1,79	**0,17±0,04**	0,16±0,03	**0,28±0,03**	18,06±2,91	7,21±3,94	0,25±0,08	3,57±0,73	0,41±0,09	0,20±0,05	**2,11±0,33**	60,20±14,30	1,14±0,22
Lyp1Δ	12,53±1,74	0,39±0,03	0,14±0,02	0,68±0,05	19,68±3,81	12,37±0,90	0,30±0,07	3,98±0,39	0,80±0,13	0,25±0,04	4,46±0,46	94,72±19,09	1,84±0,19
Gap1Δ	12,49±0,74	**0,19±0,02**	0,17±0,01	**0,45±0,04**	24,12±4,16	**2,18±0,10**	0,26±0,03	4,31±0,12	0,49±0,08	0,22±0,03	**2,12±0,19**	93,46±8,69	1,77±0,13
Mup1Δ	14,23±1,92	0,29±0,05	0,20±0,03	0,85±0,22	18,08±2,66	0,99**	0,31±0,06	5,98±0,80	0,40±0,08	**0,41±0,03**	3,33±0,36	70,15±18,02	1,26±0,18
WT	13,31±1,85	0,43±0,06	0,16±0,02	0,70±0,03	18,88±2,57	15,45±3,84	0,26±0,05	3,81±0,41	0,63±0,18	0,27±0,02	4,39±0,50	74,76±22,40	1,62±0,31

*Trp-L concentrations are in µM.
**His-L concentrations in the Mup1Δ mutant are from a single measurement.
Concentrations in bold case indicate statistically significant differences compared to wild-type (WT) strain (Student t-test $p<0.05$).
OE, overexpression; Δ, deletion.

Supplementary Table 5. Coefficients of variables for PCA analysis of extracellular and intracellular extracts.

	Intracellular		Extracellular	
	PC1	PC2	PC1	PC2
Arg-L	-0,16	0,60	0,17	0,48
Asn-L	0,35	0,07	0,30	-0,27
Asp-L	0,37	0,09	0,30	0,01
Citr-L	-0,16	0,41	0,09	-0,26
Gln-L	0,12	0,47	0,22	0,43
Glu-L	0,35	0,26	0,35	-0,33
Met-L	0,36	-0,06	0,28	0,19
Phe-L	0,37	-0,07	0,39	-0,22
Ser-L	0,11	0,40	0,34	0,31
Thr-L	0,38	-0,02	0,41	-0,28
Tyr-L	0,36	-0,11	0,30	0,26

3 References

Jessop-Fabre, M. M. *et al.* (2016) 'EasyClone-MarkerFree: A vector toolkit for marker-less integration of genes into Saccharomyces cerevisiae via CRISPR-Cas9', *Biotechnology Journal*, 11(8), pp. 1110–1117. doi: 10.1002/biot.201600147.

Nour-Eldin, H. H. *et al.* (2006) 'Advancing uracil-excision based cloning towards an ideal technique for cloning PCR fragments.', *Nucleic acids research*, 34(18), p. e122. doi: 10.1093/nar/gkl635.
